# Translational Feasibility of Curcumin for Treatment of Alzheimer’s Disease: A Critical Appraisal of Clinical Challenges

**DOI:** 10.3390/antiox15050638

**Published:** 2026-05-18

**Authors:** Jasmine Priya Virk, Malika G. Fernando, Prita Riana Asih, Ralph N. Martins

**Affiliations:** 1 Macquarie Medical School, Macquarie University, Sydney, NSW 2109, Australia; 2 Alzheimer’s Research Australia, Sarich Neuroscience Research Institute, Nedlands, WA 6009, Australia; 3 School of Medicine, University of Western Australia, Nedlands, WA 6009, Australia

**Keywords:** curcumin, Alzheimer’s disease, intranasal, nanotechnology, bioavailability, clinical translation

## Abstract

The absence of robust and effective treatments for Alzheimer’s disease remains a major challenge in modern medicine. As one of the leading causes of death, its increasing prevalence and complex chronic pathogenesis impose a substantial societal and healthcare burden, intensifying the need for effective therapeutic strategies. Current treatments remain limited, with minimal impact on cognitive decline in symptomatic patients. Curcumin, the bioactive ingredient in turmeric, has taken precedence over other natural products due to its potent antioxidative and anti-inflammatory properties. Numerous publications have extensively reported on the therapeutic effect of curcumin in animal models of Alzheimer’s disease. However, no curcumin formulation has demonstrated consistent clinical efficacy against Alzheimer’s or other neurodegenerative diseases to date. Over the years, many critics have argued that curcumin’s undesirable chemical properties, mainly low bioavailability and rapid metabolism, pose significant barriers to its therapeutic use to target the brain. Considerable funding and research effort on emerging technologies such as nanoparticles and intranasal delivery continue to drive curcumin preclinical and clinical trials, prompting reflection on the rationale for continued investment. This narrative review critically dissects this disconnect, arguing that many purported benefits remain insufficiently substantiated, and identifying important opportunities where future research may hold promise for an effective treatment.

## 1. Introduction

As the global population ages, the number of dementia cases is expected to rise to 152.8 million by 2050 [1]. Of these, approximately 60–70% are estimated to be caused by Alzheimer’s disease (AD), though this figure may be underestimated due to the disease’s diagnostic limitations and complex pathology [2]. While ineffective symptomatic drugs are being replaced with disease-modifying treatments, effectively removing pathological features like amyloid-beta (Aβ), the impact on slowing or reversing cognitive decline is limited to several months. Pathologically, AD is defined by an abnormal buildup of Aβ and neurofibrillary tangles (NFT) deposits with a specific spatial distribution in the brain, distinguishing AD from other dementias [3]. These brain deposits, while important in the pathogenesis of AD, are not solely causative of clinical symptoms such as cognitive decline, memory loss and psychiatric symptoms [3,4]. This leaves patients with a progressive decline in mental function, resulting in an increasing loss of quality of life.

Although hallmark pathological features of AD are well established, the precise aetiology remains unknown. This uncertainty complicates drug development as there are several proposed biological targets and mechanisms, and yet no approach has yielded a durable disease-modifying strategy that effectively stops the disease course. This translational gap is starkly illustrated by a 2023 systematic review which showed 141 unique treatments across 187 clinical trials to date [5]. Recently developed immunotherapies which partially slow cognitive and functional decline in early AD by clearing Aβ plaques are limited by adverse events like brain swelling and bleeding in some individuals, no improvement on survival outcomes, and a short-term benefit on cognition [6,7]. Turning to alternative mechanisms to treat AD, such as indirect modulation of NFTs and Aβ, scavenging reactive oxygen species (ROS) and promoting neurogenesis and dendritic morphology, could be of significant clinical benefit to lessen the disease burden for patients. Thus multitarget-directed ligands offer promising therapies, a strategy exemplified by the highly bioactive, natural product curcumin.

Curcumin makes up 2–9% of the spice turmeric, with recognised antioxidative and anti-inflammatory properties in Ayurvedic medicine [8]. Despite its historical use and promising multi-target profile, curcumin’s action is blunted by shortcomings. One such limitation is pan-assay interference and enzyme promiscuity, properties commonly observed in natural products [9,10]. Nelson et al. propose that curcumin exhibits activities like pan-assay interference compounds (PAINS) such as: metal chelation, redox reactivity, self-aggregation, membrane disruption, fluorescence interference and structural decomposition [10]. These properties should caution investigators when designing experiments and interpreting results with curcumin in vitro. Additionally, curcumin exhibits poor bioavailability and unfavourable absorption, distribution, metabolism and excretion properties, limiting its current use in human trials [10,11]. Although these suboptimal properties would typically rule out several lead compounds, many research groups across the world have dedicated substantial resources to address these issues due to an overwhelming number of preclinical and cellular studies which propose curcumin has significant therapeutic benefit. This narrative review will therefore critically evaluate whether the substantial investment in curcumin is justified by the evidence, separating unsupported claims from genuine therapeutic potential.

## 2. Targeting Pathophysiology of AD

### 2.1. Amyloid-β

The Aβ cascade hypothesis remains a central paradigm in AD research which posits that Aβ aggregation catalyses several downstream reactions such as oxidative stress and NFT formation [12,13]. The cascade is initiated by cleavage of amyloid precursor protein (APP) via β- and γ-secretases, producing non-toxic Aβ monomers that aggregate into toxic species such as oligomers, protofibrils, fibrils and plaques [12,13]. Emerging evidence suggests soluble protofibrils and oligomers, particularly the Aβ42 isoform, are the most synaptotoxic [14,15,16]. Soluble Aβ species have higher mobility than insoluble fibrils and can disseminate across brain regions implicated in AD pathology, including the hippocampus, entorhinal cortex, amygdala and basal forebrain [13,17]. Established evidence linking Aβ to AD pathogenesis solidifies it as a target, though the modest clinical benefits of anti-amyloid therapies have called the hypothesis’ primacy into question.

Numerous preclinical studies suggest curcumin binds to Aβ with high affinity; however, its translational relevance remains uncertain due to widespread methodological inconsistencies, leading to contradictory conclusions. Necula et al. suggest that curcumin accelerates Aβ fibril formation while inhibiting oligomerisation [18]. These results have been replicated in two independent rodent studies, indicating that aggregates may shift to less toxic species in the presence of curcumin [19,20]. In contrast, Yang et al. reported that low-dose curcumin significantly inhibited Aβ40 fibrillation and oligomerisation, while also disaggregating pre-formed fibrils in APP and Presenilin-1 (PS1) transgenic mice [21]. This study is notably limited by the lack of empirical evidence confirming blood–brain barrier (BBB) penetration. Nevertheless, this contradiction regarding curcumin’s fundamental mechanism of action highlights a lack of consensus regarding its fundamental mechanism of action. Other mechanisms suggest that curcumin significantly reduces Aβ monomer oligomerization [18,20,22,23]. These mechanistic studies suggest two co-occurring mechanisms: (1) curcumin shifts aggregates to a non-toxic pathway; (2) curcumin inhibits monomer aggregation. Tang et al. puts forward a molecular docking and nuclear magnetic resonance study (a validated methodology for PAINS) which supports the former conclusion, indicating that curcumin’s polar hydroxyl group destabilises β-sheets by binding to polar pockets in Aβ peptides [24,25].

These studies are fundamentally limited by their ability to simulate physiological conditions, either using expression models or Aβ-pharmacologically induced models. To date, there are no published curcumin studies in humanised or knock-in AD models which recapitulate physiological AD pathogenesis. In addition, most studies failed to quantify curcumin metabolites, use detergent-insoluble Aβ controls or apply orthogonal imaging modalities. Without these controls, curcumin’s therapeutic significance remains unresolved, limiting confidence in clinical translation [10]. While many limitations previously described could be resolved in future studies, many of these shortcomings reflect the inability to replicate Aβ pathogenesis in vitro, common across the current research landscape. These studies provide rationale for further preclinical and clinical investigations despite these limitations, although caution is advised when highlighting the translational significance of these results.

### 2.2. Tau

Tau deposits are hallmark pathological features of AD resulting from hyperphosphorylated tau, an intrinsically disordered microtubule-associated protein which alters microtubule dynamics during axonal transport and neuron development [4,26,27]. Pathological tau, in its hyperphosphorylated form, accumulates in stages to form NFTs: (1) initial cytoplasmic accumulation (pre-NFTs); (2) formation of dense filamentous aggregates displace the nucleus (mature NFTs); (3) degeneration of the host neuron, leaving behind extracellular ‘ghost’ tangles [26]. Although inter-individual variability in tau hyperphosphorylation is well documented, Aβ aggregation may accelerate this mechanism due to activation of kinases such as cyclin-dependent kinase 5 (CDK5) and glycogen synthase kinase-3 beta (GSK-3β) [17,28]. Due to this association between tau and Aβ, Zhang et al. suggest that targeting GSK-3β could help in diminishing aggregate deposition, highlighting the potential value of curcumin [28,29,30]. The translational feasibility of this mechanism remains to be proven.

In vitro studies have shown that curcumin binds to the microtubule-associated regions of tau, promoting tau disaggregation and reducing the NFT diameter [31]. This is corroborated by in vivo and ex vivo studies which either demonstrate weak binding affinity [32], or indirect inhibition via molecular chaperones and the CDK5 pathway [33,34]. As the strength of curcumin’s binding affinity to NFTs is highly disputed, especially as Mutsuga et al. highlight methodological issues with curcumin staining techniques, investigating curcumin’s role in indirect inhibition of tau pathology may prove more promising [32]. A glioblastoma study hinted at this broader role of curcumin in heat shock protein (HSP) regulation, though its relevance to neuronal tauopathy is highly speculative due to the model used [35]. These findings must be interpreted with caution due to curcumin’s propensity for assay interference, as their direct relevance to NFT clearance in the brain is uncharacterised. HSPs are known to contribute to microtubule stability, protecting against tauopathies [33,36]. The relationship between curcumin, HSPs and tau in AD models remains unvalidated, representing a fundamental gap which must be addressed before this mechanism can be considered clinically relevant.

### 2.3. Clinical Evidence

Clinical evidence for curcumin’s effects on AD biomarkers is difficult to interpret due to heterogeneous study populations, methodologies, delivery systems, and curcumin formulations, leading to inconsistent findings. A summary of the main methodology and outcomes is shown in Table 1 to highlight these differences. Ringman et al. report no significant effect on Aβ and tau clearance, in contrast to DiSilvestro et al. and Das et al., who report significant reductions [37,38,39]. These discrepancies may also reflect differences in methods used to assess the clearance of these biomarkers. Baum et al. report mixed results, indicating increased serum Aβ clearance despite low curcumin metabolite concentrations [40]. However, the interpretation of elevated serum Aβ remains controversial, with proposed explanations including increased clearance, increased amyloid burden, or equilibrium between cerebrospinal fluid (CSF) and plasma compartments [41,42,43]. As such, additional biomarkers, including CSF protein levels and positron emission tomography (PET) imaging, should be used to confirm target engagement [44].

The certainty of these findings is further limited by methodological shortcomings, including the omission of *p*-values and overlapping error bars, as reported in DiSilvestro et al., who observed a significant decrease in Aβ and tau following one month of oral curcumin administration [38]. Although Das et al. report similar findings, their study is limited by small sample size and the lack of independent replication [39]. While these results suggest that curcumin may influence AD biomarkers, the current body of clinical evidence remains insufficient to support this conclusion.

Other studies suggest that curcumin may exert indirect effects on AD pathology [45,46]. Small et al. demonstrated that curcumin reduced FDDNP binding in the amygdala, with no significant increase in the hypothalamus, whereas the placebo group showed increases in both regions of interest [45]. Although these findings suggest an effect on AD biomarkers, the authors note that due to low BBB penetration and limited cognitive improvement, curcumin may act through alternative mechanisms. Thota et al. report that curcumin reduces GSK-3β levels, a kinase implicated in tau and Aβ pathogenesis [46]. This pathway may partially explain curcumin’s proposed therapeutic effects and warrants further investigation in human trials, as supported by preclinical studies [28,29].

Compared to Aβ-targeting agents such as bapineuzumab, solanezumab, and crenezumab, or tau inhibitors such as TRx0237 [47,48], curcumin’s clinical evidence appears comparatively limited. Curcumin should be evaluated against the same standards as other lead compounds to ensure that more effective therapies can be developed. However, several barriers remain, particularly its limited bioavailability, which continues to constrain its clinical applicability.

**Table 1:** Clinical trial summary table.

Study	Study Design	Setting	Population Condition	Randomised (n)	Study Groups	Intervention/s	Duration	Primary Outcome
Abdolahi et al. 2017 [49]	RCT: Double blind	University; Iran	Adults with episodic migraines	ω-3 fatty acids and nano-curcumin: n = 17; ω-3 fatty acids: n = 19; Nanocurcumin: n = 19; Placebo: n = 19	ω-3 fatty acids and nano-curcumin: n = 17; 82% female, 35.8 mean age; ω-3 fatty acids: n = 19; 88% female, 36.2 mean age; Nanocurcumin: n = 19; 88% female, 37.4 mean age; Placebo: n = 19; 88% female, 36.6 mean age	ω-3 fatty acids 2500 mg in 2 capsules and 80 mg nano-curcumin in 1 capsule once daily; ω-3 fatty acids 2500 mg in 2 capsules and paraffin oil placebo in 1 capsule once daily; nano-curcumin 80 mg in 1 capsule and paraffin oil in 2 capsules once daily; paraffin oil placebo in 2 capsules once daily	56 days	Anti-inflammatory and neuroprotective effects of nano-curcumin and ω-3 fatty acids.
Baum et al. 2008 [40]	RCT: Double blind	Clinic; China	Adults (50 years and older), ethnically Chinese, mild cognitive decline and memory loss with suspected AD	1 g curcumin: n = 11; 4 g curcumin: n = 11; Placebo: n = 12	1 g curcumin: n = 8; 88% female, 77.8 mean age; 4 g curcumin: n = 11; 73% female, 73.4 mean age; Placebo: n = 8; 63% female, 69.0 mean age	1 g curcumin powder and 3 g colour matched placebo powder once daily either as capsules or powder with food; 4 g colour matched placebo powder once daily either as capsules or powder with food	180 days	Examining safety, biochemical and cognitive effects of long-term curcumin supplementation.
Cox et al. 2020 [50]	RCT: Double blind	Clinic; Australia	Healthy adults (50–85 years old)	Curcumin: n = 46; Placebo: n = 43	Curcumin: n = 39; 50% female, 67.8 mean age; Placebo: n = 40; 42.2% female, 68.4 mean age	Longvida^©^ 400 mg capsule once daily in morning; dextrin placebo capsule once daily in morning	84 days	Feasibility of clinical translation and reflection of preclinical results.
Das et al. 2023 [39]	RCT: Double blind	Clinic; India	Moderate dementia due to AD onset	USC: n = 16; CGM: n = 16; Placebo: n = 16	USC: n = 15; 38% female, 62.8 mean age; CGM: n = 16; 24% female, 64.9 mean age; Placebo: n = 13; 31% female, 66.1 mean age	South Indian diet and 3 g of 400 mg Curcumin-galactomannan complex or unformulated standard curcumin mixed with water; South Indian diet and 400 mg microcrystalline cellulose (with 250 ppm turmeric oil) once daily	182 days	Effect of curcumin on moderate dementia cognitive symptoms.
den Haan et al. 2022 [51]	Cohort; Single blind	Research centre; England	AD patients and healthy controls	AD: n = 26; Control: n = 14	AD: n = 26; 38% female; 67 mean age; Control: n = 14; 71.4% female, 71 mean age	Longvida 400 mg oral dose daily **; Theracurcumin 180 mg oral dose daily ***; Novasol 300 mg and 500 mg oral dose daily ****	10 days	To visualise retinal amyloid using curcumin.
DiSilvestro et al. 2012 [38]	Clinical Trial	N/A	Healthy adults (40–60 years old)	N/A	Curcumin: n = 19, 89% female, 48 mean age; Placebo: n = 19, 89% female, 47 mean age	Longvida^®^ 400 mg capsule once daily; starch placebo capsule once daily	28 days	Whether curcumin could alter wellness-related measures in health adults.
Fança-Berthon et al. 2021 [52]	RCT; Open label	Research centre; France	Healthy adults (18–45 years old)	Study group: n = 30	Study group *****: n = 30; 53% female, 33.6 mean age	1500 mg standard turmeric extract once; 100 mg liquid micellar preparation once; 1515 mg piperine-curcuminoid combination once; 1000 mg phytosome formulation once; 300 mg dried colloidal suspension once	90 days	Assess pharmacokinetics of turmeric in different formulations.
Kuszewski et al. 2020 [53]	RCT: Double blind	Research centre; Australia	Overweight adults (50–80 years old; BMI 25–40)	Fish oil: n = 38; Curcumin: n = 38; Fish oil and curcumin: n = 38; Placebo: n = 38	Fish oil: n =32; 53% female, 65.8 mean age; Curcumin: n = 31; 52% female, 657 mean age; Fish oil and curcumin: n = 31; 55% female, 66.1 mean age; Placebo: n = 32; 56% female, 65.8 mean age	Fish oil 400 mg in 4 capsules and 2 placebo capsules once daily; Longvida^®^ 800 mg in 2 capsules and 4 placebo capsules once daily; Fish oil 400 mg in 4 capsules and Longvida^®^ 800 mg in 2 capsules once daily; 6 placebo capsules once daily	112 days	Effects on cognitive performance and cerebrovascular responsiveness to cognitive stimuli.
Laksmidewi et al. 2024 [54]	RCT: Double blind	Hospital; Indonesia	Cervical cancer patients undergoing chemotherapy	Curcumin: n = 39; Placebo: n = 39	Curcumin: n = 39; 100% female, 46.7 mean age; Placebo: n = 39; 100% female, 50.5 mean age	Curcumin 60 mg in 3 caplets four times daily; placebo in 3 caplets for four times a day	210 days	Evaluate the safety profile of curcumin and if curcumin administration can maintain cognitive function.
Panahi et al. 2015 [55]	RCT: Double blind	Hospital; Iran	Adults with metabolic syndrome	Curcumin-piperine: n = 59; placebo: n = 58	Curcumin-piperine: n = 50; 46% female, 44.8 mean age; Placebo: n = 50; 54% female, 43.5 mean age	Curcumin C3 Complex^®^ (with 5 mg piperine) 500 mg twice daily; placebo twice daily	56 days	Measuring curcumin’s effect on oxidative stress and inflammation.
Rainey-Smith et al. 2016 [56]	RCT: Double blind	Research centre; Australia	Healthy adults (40–90 years old)	Curcumin: n = 80; Placebo: n = 80	Curcumin: n = 39; 66.7% female, 67.2 mean age; Placebo: n = 57; 73.7% female, 65.2 mean age	Biocurcumax^TM^ 500 mg in capsules three times daily after meals with water; placebo capsule 3 times daily after meals with water	364 days	Ability of curcumin to prevent cognitive decline in older adults.
Ringman et al. 2012 * [37]	RCT; Double blind	Research centre; USA	Mild to moderate AD	2 gm Curcumin: n = 12; 4 gm Curcumin: n = 12; Placebo: n = 12	2 gm Curcumin: n = 9; 67% female; 76.7 mean age; 4 gm Curcumin: n = 10; 70% female; 75.3 mean age; Placebo: n = 11; 55% female; 70.2 mean age	2 gm Curcumin C3 Complex^®^ in 4 capsules, once daily; 4 gm Curcumin C3 Complex^®^ in 4 capsules, once daily; 500 mg placebo in ×4 capsules once daily	336 days	Incidence of adverse events, changes in clinical laboratory tests and the ADAS-Cog at 24 weeks.
Santos-Parker et al. 2017 [57]	RCT: Double blind	Research centre; USA	Healthy adults (45–74 years old)	Curcumin: n = 23; Placebo: n = 21	Curcumin: n = 20; 50% female, 63 mean age; Placebo: n = 19; 42% female, 61 mean age	Longvida^®^ 2000 mg capsules once daily; placebo capsules once daily	84 days	Preclinical to clinical translation of improved age related vascular endothelial function.
Small et al. 2018 [45]	RCT: Double blind	Medical centre; USA	Non-demented adults	Curcumin: n = 23; Placebo: n = 23	Curcumin: n = 21; 57% female, 63.1 mean age; Placebo: n = 19; 53% female sex, 62.9 mean age	Theracurmin^®^ 90 mg twice daily; placebo twice daily	546 days	Curcumin’s anti-inflammatory and neuroprotective properties effect on neurodegeneration.
Thota et al. 2020 [46]	RCT: Double blind	Medical centre; Australia	Adults with high risk of type 2 diabetes (30–70 years old)	Placebo: n = 19; Curcumin: n = 20; Fish oil: n = 20; Curcumin + fish oil: n = 22	Placebo: n = 16; 56% female, 50 mean age; Curcumin: n = 15; 60% female, 55 mean age; Fish oil: n = 17; 59% female, 58 mean age; Curcumin + fish oil: n = 16; 63% female, 57 mean age	21,000 mg corn oil, 2 placebo tablets matching curcumin capsules once daily; 2500 mg Meriva^®^ tablets containing 180 mg and 21,000 mg corn oil capsules once daily; 21,000 mg fish oil capsules and 2 placebo tablets matching curcumin once daily; 2500 mg Meriva^®^ tablets containing 180 mg curcumin and 21,000 mg fish oil capsules once daily	84 days	Evaluate the effect of curcumin and or fish oil on parameters relating to glucose.

RCT = Randomised clinical trial. AD = Alzheimer’s disease. * Participants in the placebo group were 1:1 randomly assigned to either the 2 gm or 4 gm group at 24 weeks. ** Intervention on 14 AD patients and 12 controls for 10 days. *** Intervention on 7 AD patients and 2 controls for 5 days. **** Intervention on 5 AD patients, 300 mg for 4 days and 500 mg for last day, ***** Cross-over study.

## 3. Curcumin’s Antioxidative Properties

Oxidative stress (OS) is characterised by an excess of ROS which overwhelm endogenous antioxidant defences, contributing to cellular damage, neuron death and lipid peroxidation, playing a key role in the pathophysiology of ageing [58,59]. Keller et al. posit that OS may be one of the earliest events in onset and progression of AD in their clinical study revealing that elevated levels of oxidants and evidence of OS precedes AD diagnosis in adults with mild cognitive decline [59]. This is further strengthened by post-mortem analysis, suggesting that oxidative damage is often specific and highest in neuroanatomy which is implicated in AD (such as the prefrontal cortex and hippocampus) [59,60]. Despite the strong theoretical rationale for antioxidants in AD, the clinical evidence for curcumin’s antioxidative efficacy is marked by inconsistency.

The mechanisms of action for curcumin’s antioxidative properties are not fully elucidated. Currently, strong clinical evidence proposes that curcumin improves antioxidant activity as well as decreasing burden of OS [55,57]. Curcumin is thought to scavenge very reactive oxygen species (OH, NO_2_), peroxides (such as H_2_O_2_) and metals/metal oxides (Fe_2_O_3_, Cd, Pb, Cu), scavenging ROS through electron transfer or hydrogen abstraction [8,24,61]. A chemical study using pulse radiolysis and laser flash photolysis describe how curcumin eliminates lipid radicals from the cell [62]. The authors suggest that curcumin positions itself within the cell membrane, intercepting lipid radicals and pushing the radicals, due to its polarity, to the surface of the membrane where it will be then neutralised by water-soluble antioxidants [62].

These elaborate mechanisms are often demonstrated in purified, sterile chemical systems, and remain to be validated in the context of curcumin’s rapid metabolism and low bioavailability. Ringman et al. show that curcumin supplementation has no effect on F2-isoprostanes [37], a validated method for measuring OS in other clinical trials [63]. Cox et al. supports these findings showing that a different curcumin formulation (see Table S1) did not alter OS measures such as 8 hydroxy deoxyguanosine or protein carbonyls [50]. Alternatively, DiSilvestro et al. shows that patients who took the same dose and formulation of curcumin to Cox et al., but with an Indian diet, showed significantly lower levels of myeloperoxidase and nitric oxide [38]. This discrepancy may be due to differences in choices of oxidative biomarkers or more interestingly, the addition of an Indian diet which may lead to increased curcumin bioavailability, as validated in the literature [64].

Unlike curcumin’s other antioxidative properties, its chelating effect is well characterised [61]. However, its benefit is disputed as metal-curcumin complexes are positively charged, binding to negatively charged DNA and inducing damage [65,66]. Priyadarsini et al. suggest that the bioavailability of metal-curcumin is so low that any damage would be insignificant [8], highlighting a discrepancy between theoretical and clinical translation. This claim is backed by a clinical trial where curcumin was seen to have a therapeutic effect on metal prooxidative DNA damage [67]. This evidence could be adapted to a current hypothesis on iron homeostasis driving OS in AD, especially since curcumin’s hydroxyl and methoxy group are strong iron chelators [61]. Considering this dispute, like other curcumin properties, future investigations should consider the influence of biological variables on curcumin’s therapeutic or toxic effects.

## 4. Regulating Neuroinflammation

It has been long recognised that neuroinflammation plays a major role in the pathogenesis of AD; however, it is yet to be determined whether neuroinflammation precedes clinical symptoms or is a consequence of this disease [68]. Many studies have shown that OS, NFTs and Aβ have an effect on expression and activation of immunomodulatory cytokines released by microglia and astrocytes such as interleukin-1 (IL-1), nuclear factor kappa B (NFκB), tumour necrosis factor alpha (TNF-α) and nuclear factor erythroid 2–related factor 2 (Nrf2) [30,49,69,70,71,72,73]. This leads to cell death, causing further excitation of proinflammatory cytokines, therefore leading to a positive feedback loop [74]. These immune responses may be exacerbated by genetic factors, such as polymorphisms in IL-1, TNF-α and Triggering receptor expressed on myeloid cells 2 (TREM2) which are associated with cytokines and microglial inflammation [75,76].

Astrocytes, when activated, produce inflammatory mediators which are closely linked to AD. One prominent mediator is glial fibrillary acidic protein (GFAP), a common marker to reflect activated astrocytes under elevated inflammatory conditions [72,77]. Preclinical studies are divided on whether curcumin activates or inhibits GFAP. However, these two opposing effects may be explained by differences in curcumin dose. Lim et al. found that low-dose curcumin decreased GFAP by 16.5%, consistent with the findings of Ambegaokar et al. and Zhang et al. [72,78,79]. In contrast, Seady et al. found that high-dose curcumin alters actin networks in astrocytes, increasing GFAP activity [80]. These latter findings argue for a dose-dependent effect of curcumin on GFAP levels.

Clinical evidence has provided mixed results, partially due to measurement of different markers of inflammation. The findings of Das et al. demonstrate mixed results, showing a significant decrease in TNF- α but not IL-9 [39]. It should be noted these results may be due to the lack of power from differences in sample size and may be influenced by gender demographic differences. DiSilvestro et al. observed a significant increase in plasma myeloperoxidase concentration not accompanied by an increase in inflammatory markers such as C reactive protein, inferring curcumin could increase immune regulation while not triggering an inflammatory response [38]. These claims should be validated in future clinical trials. Curcumin’s anti-inflammatory effect is yet to be fully characterised in neurodegenerative disease models.

## 5. Neurogenesis and Dendritic Plasticity

The potential role of neurogenesis in AD could prove revolutionary, redefining AD management to focus on neuron regeneration in addition to inhibiting neurodegeneration, giving potential hope to symptomatic patients. Despite compelling preclinical evidence, typically measured with BrdU labelling, curcumin stimulation of adult neurogenesis remains elusive and controversial, requiring a significant amount of research for conclusive evidence to be demonstrated [81,82]. Preclinical trials must control various factors such as cohort demographics, BrdU administration timing and reliance on surrogate markers, making cross-study analysis challenging [81]. Several studies found that curcumin has a dose-dependent effect on neural progenitor cells (NPCs) proliferation, which populate the central nervous system (CNS) with glial and neuronal cells, through the extracellular signal-regulated kinase and p38 MAPK signalling pathway in vitro [82,83]. Both Kim et al. and Lee et al. found low-dose curcumin (0.1–2 uM) increased proliferation while high doses (>10 uM) caused a decrease, raising translational concerns [82,84]. Although neurogenic effects were observed, such as increased BrdU+ and NeuN+ cell counts in vivo, their significance will need to await behavioural tests and BBB penetration [82,83,85].

Dendritic architecture, significantly implicated in AD pathology, may be impacted by curcumin, potentially increasing neuroplasticity [16,86]. Currently, short study durations may result in false positive findings; therefore, many preclinical studies are subject to significant confounding variables [86,87]. While studies currently indicate curcumin may improve spine density, i.e., MAP2 staining, PSD95 and Homer1, further confirmatory studies are needed [85,88]. Future studies should address methodological standardisation, clarify pharmacokinetic parameters and employ the use of orthogenic controls to determine curcumin’s role in regenerating neurons.

## 6. Curcumin’s Effect on Cognition

Despite public attention and decades of research, clinical evidence for curcumin’s cognitive benefits in AD remains unconvincing. To date, most clinical trials investigating curcumin’s potential role in AD have been conducted in populations with either mild or moderate AD or mild cognitive impairment. It could be argued that the disease is too advanced in the latter groups for curcumin to have an effect. Instead, cognitively normal individuals with a high burden of neuropathology would be most likely to benefit from curcumin treatment; Small et al. and Rainey-Smith et al. both propose that future trials explore these cognitively intact high-risk populations for curcumin’s beneficial effects to be evident [45,56]. When testing 400 mg of Biocurcumax^®^ on the cognition of 96 healthy older adults in a randomised, 12-month controlled trial, Rainey-Smith et al. found there was no significant changes from baseline and follow-up within group but exhibited significant between group differences, suggesting curcumin may stabilise cognitive performance rather than modulating existing deficits [56]. Das et al., instead reported significant improvement from baseline and in group–group analysis, attributing these findings to increased bioavailability of their proprietary formulation, although this pharmacokinetics were not reported on [39].

Despite these findings, multiple studies reported no significant difference between curcumin and the placebo [37,50,53]. Ringman et al. reported that curcumin-treated patients performed worse than individuals on the placebo control in cognitive measures, although this difference was not statistically significant and has not been replicated in a controlled setting [37]. Additionally, Laksmidewi et al. and Cox et al. both noted that the curcumin group self-reported reduced perceived cognitive function, especially in the capacity to perform daily tasks, although these perceptions were not confirmed by objective cognitive assessment [50,54]. Regardless of these shortcomings, Laksmidewi et al. still reported significant improvements in selective attention, assessed by the Stroop test, highlighting curcumin’s ability to improve selective aspects of cognition [54].

Of the clinical trials included in this review, many fail to measure curcumin metabolites, report on curcumin’s pharmacokinetics and measure AD biomarkers; notably, there may be justifiable reasons for why these limitations occur. Disparate claims are also highly attributed to the diverse use of curcumin derivatives and formulations, which, although a reflection on the rapid innovation in curcuma longa development, makes replication and synthesis of data implausible.

## 7. Issues with Bioavailability and Metabolism

One of the most significant barriers to curcumin’s development as a viable Alzheimer’s therapy is its extremely poor bioavailability. This has historically limited its therapeutic translation despite promising in vitro results. Bioavailability, broadly defined as the fraction of an administered dose that reaches systemic circulation unchanged, is especially critical for CNS drugs, where BBB penetrance is essential. While some early curcumin studies indicated neuroprotective effects, these were primarily conducted under non-physiological conditions, using unrealistic incubation periods, high concentrations and pH and temperature adjusted for curcumin’s chemical properties, hence obscuring curcumin’s translational value [8,89]. Wang et al., an early study on curcumin pharmacokinetic parameters, found that 90% of curcumin was shown to degrade within 30 min in stimulated physiological conditions, raising questions about feasibility in a clinical setting [89].

Not only does curcumin’s poor metabolism impact its pharmacokinetics, but it may also influence tolerability and safety outcomes. Clinical studies report mixed findings regarding adverse events. For instance, Rainey-Smith et al. and Small et al. reported adverse event rates of approximately 14% and 15% respectively [45,56], whereas Baum et al. and Das et al. reported no treatment-related adverse events [39,40]. When reported, adverse effects are generally mild and include abdominal discomfort and transient gastrointestinal disturbances [45,56]. Differences in tolerability across studies may reflect formulation, dosing regimens, participant populations and dietary exposure to curcumin. Though curcumin is considered a relatively safe compound compared to other approved Alzheimer’s therapies, mild but frequent gastrointestinal side effects may still impact adherence, further limiting clinical potential. These findings support the development of alternate delivery pathways to bypass first-pass metabolism and therefore limited any adverse events.

Recent efforts have focused on improving delivery via nanoparticle formulations, liposomes, and emulsions. These vehicles aim to bypass extensive first-pass metabolism and act as “Trojan horses,” protecting curcumin as it traverses the BBB before releasing it within the CNS [90]. Intraperitoneal delivery in rodent models has yielded increases in brain curcumin levels and reductions in Aβ pathology [84]. However, no clinical evidence has definitively shown that orally administered curcumin reaches the CSF at therapeutic concentrations [37,51,91]. Importantly, systemic availability alone is insufficient; curcumin must not only circulate but also cross the BBB and remain stable in the CNS’s oxidative, aqueous environment. Thus, while many researchers continue investing in delivery systems, others argue that unless curcumin’s chemical instability and poor metabolism are addressed simultaneously, these efforts may be futile [10].

Even if delivery issues were overcome, curcumin’s rapid and extensive metabolism presents another formidable obstacle, undergoing reduction, conjugation and oxidation, producing a variety of metabolites [8,11]. Notably, these processes differ significantly across species. Ireson et al. demonstrated that curcumin is metabolised approximately 3.5 times faster in humans than in rats, yet rodent studies remain extensively used in preclinical pipelines [92].

Moreover, emerging evidence suggests that curcumin’s observed biological effects may not derive from the parent compound at all. Degradation products such as vanillin, an antioxidant and antimutagenic compound, have been detected in several in vitro assays [89]. These findings call into question whether the therapeutic outcomes are due to curcumin itself, or to its breakdown products and metabolites, as Wang et al. caution, the presence of vanillin and other byproducts should be carefully considered in assay interpretation [89]. This complexity challenges the design of mechanistic studies, where attributing an observed biological effect to a single, rapidly metabolised compound may be scientifically difficult to interpret. This has led to increasing focus on curcuminoid derivatives, particularly tetrahydrocurcumin (THC), which displays higher bioavailability and more stable activity in preliminary models [90]. Whether these derivatives can achieve better BBB penetration or circumvent hepatic metabolism remains a key question explored in the next section.

### 7.1. Tetrahydrocurcumin and Structural Analogues

THC is a major curcumin metabolite, generated during reduction, exhibiting improved water solubility, chemical stability and a larger therapeutic window compared to its parent compound [93,94]. While its phenolic and β-diketone functional groups contribute to its antioxidant activity, common in many radical scavenging molecules, its precise mechanism remain more elusive when compared to curcumin [93]. Compared to curcumin and other derivatives, THC exhibited the most rapid and potent inhibition of Aβ(40–42), while paradoxically, evidence also suggests it has the lowest binding affinity in cellular studies [93,94]. These findings could suggest indirect modulation of the amyloid-cascade, with higher stability than curcumin [93]. These findings are corroborated by Randino et al. who reported that THC required “guest molecules” to effectively interact with monomeric Aβ(25–30), suggesting activity may depend on microenvironmental conditions [95].

Structural studies, often used for identifying lead compounds, found that THC reduced β-sheet content (from 50% to 44.9%) and increased α-helical structures (from 15.2% to 25.5%), increasing stability and potentially stabilising non-toxic conformations in Aβ(1–42) cells [96]. THC has also demonstrated moderate cholinesterase inhibition—similar to the mechanism of most regulated AD treatments [5,97]. Arunkhamkaew et al. found that, THC led to cholinesterase inhibition which was twofold the potency of curcumin in vitro, potentially due to the absence of the methoxy groups and central double bond [97]. However, when combining THC with dihydropyrimidinone (DHPM) units (potent acetylcholinesterase inhibitors), results unexpectedly showed low inhibition, likely due to steric hindrance or presence of nitro groups which disrupt enzyme binding [97].

### 7.2. Additives and Absorption Enhancers

Formulation additives have been explored to improve curcumin’s notoriously low bioavailability. Small et al. compared the bioavailability of Theracurmin, Meriva, and GNC curcumin products in humans [45]. Theracurmin, a nanoparticle formulation of THC, demonstrated the highest serum levels, though interpretation is limited by the small sample size (n = 10) [45]. In a randomised controlled trial, Fanca-Berthon et al. administered 1500 mg of curcuminoid-rich formulations, including additives like quillaja extract, sunflower oil, and acacia gum [52]. Although no significant differences were found in the area under the curve (AUC) between formulations over 24 h, the turmeric–phospholipid complex (TPG) exhibited the highest relative absorption at a 300 mg dose. Additional formulations included 15 mg piperine (black pepper extract), a known inhibitor of hepatic glucuronidation, which may further enhance systemic curcuminoid levels, though such non-specific enzyme inhibitors raise safety concerns, such as increased serum glucose and liver damage [37,50,52,98].

For non-oral routes, chitosan-based formulations have shown particular promise. Casettari et al. reviewed the use of chitosan, a mucoadhesive polysaccharide derived from crustacean shells, in enhancing nasal drug delivery [99]. At acidic pH, chitosan is positively charged and can improve mucosal adhesion, facilitating brain targeting through nasal-to-olfactory transport [99]. While chitosan nanoparticles have been studied for insulin and peptide delivery, there remains a significant gap in optimising such systems for CNS-targeted curcuminoids. These additives should be considered for curcumin as well.

## 8. Nanotechnology in Curcumin Innovation

As previously alluded to, curcumin’s notoriously poor water solubility and rapid metabolism have prompted the development of nano formulations, an approach commonly employed to improve bioavailability of poorly soluble drugs. Nanoparticles, stable compound particles 10–10,000 nm in diameter, have become increasingly relevant as a reduction in particle size can improve efficacy, solubility and bioavailability [100]. These include liposomes, polymeric nanoparticles, dendrimers and micelles, which are all designed to overcome pharmacokinetic and physiochemical barriers [101,102]. Nanoparticles are often used on small or low bioavailable compounds to bypass the BBB typically encapsulated by a carrier, not unlike a Trojan horse, degrading once passing the barrier and activating the drug [101]. In curcumin formulations, recent evidence suggests that nanocurcumin is endocytosed by microglia, hence, in theory, inducing a greater therapeutic response [103].

Several in vitro and in vivo studies have evaluated effectiveness of nanocurcumin in AD models. Cheng et al. found that orally administered nanocurcumin was measured in plasma within 10 min, peaking in brain tissue at 20 min with a biphasic curve thereafter [102]. Additionally, there was no alteration to THC levels in the plasma or brain, as well as no improvement to curcumin’s area under the curve value [102]. Although this provides only a narrow window for curcumin, this is a significant improvement from oral curcumin which was not detectable in blood levels (similar to clinical trials) and may warrant further delivery modification such as intranasal delivery or use of additives [37,102].

The efficacy of nanocurcumin compared to oral curcumin is disputed across multiple studies; however, as the field grows, issues are addressed, and new innovations are made to amend previous shortcomings. Behavioural outcomes in nanocurcumin rats are variable, with nanocurcumin-treated rodents in radial arm maze (RAM) tests presented with improved cue memory but failed to show efficacy in working or reference memory [102]. Unexpectedly, senile plaques were fewer in curcumin treated mice but not nanocurcumin-treated mice [102]. The results from this study should be further evaluated considering the lack of transparency around effect size and correction for multiple analysis. Barbara et al. expands upon these results, showing that while unloaded curcumin may have promoted Aβ aggregation, glycosylated peptide (G7) loaded nanocurcumin significantly inhibited fibrillation while promoting disaggregation in group–group comparison and baseline within group comparison [104]. The authors speculated that NCs initially attract Aβ due to hydrophobic surface interactions and, with long-term adherence, destabilise fibrils through conformational rearrangements [104]. Still, the synaptic rescue observed was not significant, and the study lacked redox or fluorescence-based interference controls, which is critical when assessing curcumin’s mechanism of action. Without mechanistic insights, it is difficult to confirm the specificity of curcumin’s action.

Currently, curcumin clinical trials are evolving to test the effects of nanocurcumin, a clinical trial examining nanocurcumin in migraine patients reporting reductions in TNF-α mRNA and protein levels [49]. Despite this finding, its relevance in neurodegenerative pathology remains to be determined. While nanocurcumin formulations clearly offer improved pharmacokinetic improvements and show preliminary potential, several issues remain: many studies lack rigorous in vivo validation, ignore curcumin’s chemical instability and do not incorporate valid controls for assay interference.

### Intranasal Delivery

The potential of intranasal drug delivery to bypass the hepatic first-pass effect and facilitate direct nose-to-brain transport has sparked considerable interest for compounds with poor oral bioavailability, such as curcumin. As a lipophilic molecule with extensive first-pass metabolism, curcumin appears an ideal candidate for intranasal administration. Yet a closer examination of the mechanistic and pharmacokinetic landscape reveals both opportunities and obstacles. The nasal route offers multiple potential conduits for brain access: the olfactory neural pathway (via olfactory neurons and epithelial cells), the trigeminal pathway, and indirect systemic absorption followed by BBB penetration [99]. Lipophilic small molecules, like morphine and curcumin, exhibit more favourable nasal absorption profiles compared to peptides or hydrophilic drugs, with bioavailability exceeding 75% in some cases [99]. However, enhancing absorption through epithelial tight junctions, normally 3.9–8.4 Å in diameter, expandable to ~15 nm with enhancers, remains essential for sufficient brain delivery [99].

Despite bypassing hepatic metabolism, curcumin faces another metabolic challenge: elevated cytochrome P450 (CYP450) activity in the nasal mucosa. The olfactory region exhibits CYP450 enzyme expression even higher than the liver [99]. Given that curcumin is both a substrate and a modulator of multiple CYP450 isoforms, the enzymatic fate of curcumin in the nasal cavity remains poorly understood and underexplored despite its critical implications for dose retention, pharmacokinetics, and efficacy [10,11]. This represents a significant gap in the literature warranting urgent investigation.

Duan et al. evaluated nanocurcumin in a murine model of intracerebral haemorrhage, noting that intranasal delivery achieved delayed but sustained accumulation in the brain relative to intravenous (IV) administration [103]. While intravenous administration led to higher early concentrations due to BBB disruption, intranasal curcumin surpassed intravenous levels at 12 h and remained stable thereafter, potentially reflecting prolonged mucosal absorption and steady neuronal transport [103]. Importantly, fluorescence imaging revealed reduced curcumin distribution in peripheral tissues (e.g., liver, kidney), suggesting intranasal delivery may minimise systemic exposure and off-target effects, one of the main limitations in previous curcumin trials [103]. Mechanistically, Duan et al. also demonstrated significant epithelial penetration, with curcumin adhering to the nasal mucosa for up to 3 h, likely using intercellular or neuronal pathways [103]. Functional benefits included improved neurobehavioral outcomes and reduced microglial activation in intranasal-treated mice. These findings support curcumin’s therapeutic promise in neuroinflammatory contexts beyond acute haemorrhage, such as AD. Other studies directly investigate curcumin’s preventative and therapeutic treatment of AD preclinical mouse models. McClure et al. found that after 6 months of intranasal curcumin treatment, transgenic-treated mice performed similarly to wild-type mice in a Y-maze test, unlike the negative control transgenic mice whose results were consistent with AD pathology [105]. Feng et al. similarly shows an insignificant difference in MWM between wild-type mice and curcumin-treated transgenic mice, who were also subject to 6 months of intranasal nanocurcumin treatment [106]. Both studies validated their findings with immunohistochemistry, showing that the treated mice had decreased Aβ plaque burden [105,106,107]. It should be noted that the studies focused primarily on preventative treatment, although Feng et al. put forward data suggesting increased Aβ clearance via microglia regulation [106]. These studies present some of the most compelling data for intranasal curcumin but are yet to be translated into clinical data.

Encapsulation efficiency is critical for intranasal nanoparticles, with >80% considered necessary for therapeutic viability [103]. Formulation strategies must also contend with curcumin’s stability; for example, Vaz et al. showed that curcumin and quercetin-loaded nano emulsions were stable for one month at 4 °C, but degraded at higher temperatures [90]. Mucoadhesive designs have been proposed to increase nasal residence time and improve anterior deposition, with one abandoned patent targeting 75% anterior nasal delivery, well above the <10% achieved via passive olfaction [108]. Aerosol and dry-powder formulations have also been proposed. Aggarwal patented an aerosolized curcumin design using lipid vesicles to enhance airborne stability [109]. However, less than 10% of inspired air typically reaches the olfactory slit, limiting passive mucosal delivery unless specifically engineered to overcome this barrier [108].

Despite promising in vitro permeation and in vivo retention, the current literature remains heavily pharmaceutics focused. Most studies neglect key pharmacodynamic endpoints such as target engagement, cell signalling, or validation via orthogonal assays. The absence of robust in vivo AD models, long-term toxicity profiles, and chemical integrity analyses further weakens translational claims. Moreover, the therapeutic relevance of curcumin retention in nasal mucosa versus active neuroprotection remains to be established.

The successful intranasal delivery of drugs like carbamazepine, a lipophilic agent with low oral bioavailability, further validates the theoretical applicability of intranasal curcumin. Innovations in carbamazepine delivery, such as mucoadhesive and targeted nanocarriers, may serve as useful analogues in guiding future curcumin designs [108]. As of now, there are no completed human clinical trials specifically testing intranasal curcumin in AD. Most intranasal curcumin studies remain preclinical, thus safety and efficacy profiles for intranasal curcumin in AD patients remain incompletely characterised.

## 9. Conclusions

The narrative of curcumin as a promising multi-target therapeutic for Alzheimer’s disease is compelling in theory but remains insufficiently supported by robust clinical evidence. This review systematically demonstrates that the transition from compelling in vitro mechanisms to tangible human benefit warrants further investigation to overcome current challenges.

The promise of curcumin’s antioxidative, anti-inflammatory, and neurogenic properties is critically undermined by its profound pharmacokinetic failures; namely, negligible oral bioavailability, rapid systemic metabolism, and an inability to reliably reach the brain at therapeutic concentrations. While innovative formulations and delivery routes attempt to bridge this gap, they have yet to demonstrate consistent success in overcoming these current barriers.

Comparative evidence suggests that other structurally related natural compounds may exhibit more favourable translational profiles, highlighting the importance of benchmarking curcumin against alternative candidates rather than evaluating it in isolation.

Future research resources would be more judiciously allocated toward investigating the more stable and bioavailable metabolites of curcumin, such as THC, or toward other compounds with clearer translational pathways. Unlike curcumin with its strong yellow colour, THC is colourless, which offers an advantage over curcumin specifically for intranasal delivery. In conclusion, while the curcumin saga offers valuable lessons in drug development and the perils of extrapolating from simplistic models, the evidence points to the field moving beyond the parent compound to more promising avenues.

## Data Availability

No new data were created or analysed in this study. Data sharing is not applicable to this article.

## Supplementary Materials

The following supporting information can be downloaded at: https://www.mdpi.com/article/10.3390/antiox15050638/s1, Table S1: Preclinical trial summary table.

## References

[1] (2022). GBD 2019 Dementia Forecasting Collaborators. Estimation of the global prevalence of dementia in 2019 and forecasted prevalence in 2050: An analysis for the Global Burden of Disease Study 2019. Lancet Public Health.

[2] (2024). 2024 Alzheimer’s disease facts and figures. Alzheimer’s Dement..

[3] DeTure M.A., Dickson D.W. (2019). The neuropathological diagnosis of Alzheimer’s disease. Mol. Neurodegener..

[4] McKhann G.M., Knopman D.S., Chertkow H., Hyman B.T., Jack C.R., Kawas C.H., Klunk W.E., Koroshetz W.J., Manly J.J., Mayeux R. (2011). The diagnosis of dementia due to Alzheimer’s disease: Recommendations from the National Institute on Aging-Alzheimer’s Association workgroups on diagnostic guidelines for Alzheimer’s disease. Alzheimer’s Dement..

[5] Cummings J., Zhou Y., Lee G., Zhong K., Fonseca J., Cheng F. (2023). Alzheimer’s disease drug development pipeline: 2023. Alzheimer’s Dement. Transl. Res. Clin. Interv..

[6] Ebell M.H., Barry H.C., Baduni K., Grasso G. (2024). Clinically Important Benefits and Harms of Monoclonal Antibodies Targeting Amyloid for the Treatment of Alzheimer Disease: A Systematic Review and Meta-Analysis. Ann. Fam. Med..

[7] Zimmer J.A., Ardayfio P., Wang H., Khanna R., Evans C.D., Lu M., Sparks J., Andersen S., Lauzon S., Nery E.S.M. (2025). Amyloid-Related Imaging Abnormalities With Donanemab in Early Symptomatic Alzheimer Disease: Secondary Analysis of the TRAILBLAZER-ALZ and ALZ 2 Randomized Clinical Trials. JAMA Neurol..

[8] Priyadarsini K.I. (2014). The Chemistry of Curcumin: From Extraction to Therapeutic Agent. Molecules.

[9] Kocahan S., Doğan Z. (2017). Mechanisms of Alzheimer’s Disease Pathogenesis and Prevention: The Brain, Neural Pathology, N-methyl-D-aspartate Receptors, Tau Protein and Other Risk Factors. Clin. Psychopharmacol. Neurosci..

[10] Nelson K.M., Dahlin J.L., Bisson J., Graham J., Pauli G.F., Walters M.A. (2017). The Essential Medicinal Chemistry of Curcumin. J. Med. Chem..

[11] Priyadarsini K.I. (2009). Photophysics, photochemistry and photobiology of curcumin: Studies from organic solutions, bio-mimetics and living cells. J. Photochem. Photobiol. C-Photochem. Rev..

[12] Karran E., Mercken M., Strooper B.D. (2011). The amyloid cascade hypothesis for Alzheimer’s disease: An appraisal for the development of therapeutics. Nat. Rev. Drug Discov..

[13] Chen G.-F., Xu T.-H., Yan Y., Zhou Y.-R., Jiang Y., Melcher K., Xu H.E. (2017). Amyloid beta: Structure, biology and structure-based therapeutic development. Acta Pharmacol. Sin..

[14] De S., Wirthensohn D.C., Flagmeier P., Hughes C., Aprile F.A., Ruggeri F.S., Whiten D.R., Emin D., Xia Z., Varela J.A. (2019). Different soluble aggregates of Aβ42 can give rise to cellular toxicity through different mechanisms. Nat. Commun..

[15] Lord A., Englund H., Söderberg L., Tucker S., Clausen F., Hillered L., Gordon M., Morgan D., Lannfelt L., Pettersson F.E. (2009). Amyloid-β protofibril levels correlate with spatial learning in Arctic Alzheimer’s disease transgenic mice. FEBS J..

[16] Lacor P.N., Buniel M.C., Furlow P.W., Sanz Clemente A., Velasco P.T., Wood M., Viola K.L., Klein W.L. (2007). Aβ Oligomer-Induced Aberrations in Synapse Composition, Shape, and Density Provide a Molecular Basis for Loss of Connectivity in Alzheimer’s Disease. J. Neurosci..

[17] Hampel H., Hardy J., Blennow K., Chen C., Perry G., Kim S.H., Villemagne V.L., Aisen P., Vendruscolo M., Iwatsubo T. (2021). The Amyloid-β Pathway in Alzheimer’s Disease. Mol. Psychiatry.

[18] Necula M., Kayed R., Milton S., Glabe C.G. (2007). Small Molecule Inhibitors of Aggregation Indicate That Amyloid β Oligomerization and Fibrillization Pathways Are Independent and Distinct*. J. Biol. Chem..

[19] Caesar I., Jonson M., Nilsson K.P.R., Thor S., Hammarström P. (2012). Curcumin Promotes A-beta Fibrillation and Reduces Neurotoxicity in Transgenic Drosophila. PLoS ONE.

[20] Hamaguchi T., Ono K., Murase A., Yamada M. (2009). Phenolic Compounds Prevent Alzheimer’s Pathology through Different Effects on the Amyloid-β Aggregation Pathway. Am. J. Pathol..

[21] Yang F., Lim G.P., Begum A.N., Ubeda O.J., Simmons M.R., Ambegaokar S.S., Chen P.P., Kayed R., Glabe C.G., Frautschy S.A. (2005). Curcumin Inhibits Formation of Amyloid β Oligomers and Fibrils, Binds Plaques, and Reduces Amyloid In Vivo*. J. Biol. Chem..

[22] Rao P.P.N., Mohamed T., Teckwani K., Tin G. (2015). Curcumin Binding to Beta Amyloid: A Computational Study. Chem. Biol. Drug Des..

[23] Chiorcea-Paquim A.-M., Mascini W.B.S., Oliveira-Brett A.M. (2025). Amyloid–ß peptides interaction with curcumin: AFM and electrochemical characterisation. Electrochim. Acta.

[24] Tang M., Taghibiglou C. (2017). The Mechanisms of Action of Curcumin in Alzheimer’s Disease. J. Alzheimer’s Dis..

[25] Sun J., Zhong H., Wang K., Li N., Chen L. (2021). Gains from no real PAINS: Where ‘Fair Trial Strategy’ stands in the development of multi-target ligands. Acta Pharm. Sin. B.

[26] Metaxas A., Kempf S.J. (2016). Neurofibrillary tangles in Alzheimer’s disease: Elucidation of the molecular mechanism by immunohistochemistry and tau protein phospho-proteomics. Neural Regen. Res..

[27] Mandelkow E.-M., Mandelkow E. (2012). Biochemistry and Cell Biology of Tau Protein in Neurofibrillary Degeneration. Cold Spring Harb. Perspect. Med..

[28] Zhang H., Wei W., Zhao M., Ma L., Jiang X., Pei H., Cao Y., Li H. (2021). Interaction between Aβ and Tau in the Pathogenesis of Alzheimer’s Disease. Int. J. Biol. Sci..

[29] Lou S., Gong D., Yang M., Qiu Q., Luo J., Chen T. (2024). Curcumin Improves Neurogenesis in Alzheimer’s Disease Mice via the Upregulation of Wnt/β-Catenin and BDNF. Int. J. Mol. Sci..

[30] Bustanji Y., Taha M.O., Almasri I.M., Al-Ghussein M.A.S., Mohammad M.K., Alkhatib H.S. (2009). Inhibition of glycogen synthase kinase by curcumin: Investigation by simulated molecular docking and subsequent in vitro/in vivo evaluation. J. Enzym. Inhib. Med. Chem..

[31] Rane J.S., Bhaumik P., Panda D. (2017). Curcumin Inhibits Tau Aggregation and Disintegrates Preformed Tau Filaments in vitro. J. Alzheimer’s Dis..

[32] Mutsuga M., Chambers J.K., Uchida K., Tei M., Makibuchi T., Mizorogi T., Takashima A., Nakayama H. (2012). Binding of Curcumin to Senile Plaques and Cerebral Amyloid Angiopathy in the Aged Brain of Various Animals and to Neurofibrillary Tangles in Alzheimer’s Brain. J. Vet. Med. Sci..

[33] Ma Q.-L., Zuo X., Yang F., Ubeda O.J., Gant D.J., Alaverdyan M., Teng E., Hu S., Chen P.-P., Maiti P. (2013). Curcumin Suppresses Soluble Tau Dimers and Corrects Molecular Chaperone, Synaptic, and Behavioral Deficits in Aged Human Tau Transgenic Mice*. J. Biol. Chem..

[34] Sundaram J.R., Poore C.P., Sulaimee N.H.B., Pareek T., Cheong W.F., Wenk M.R., Pant H.C., Frautschy S.A., Low C.-M., Kesavapany S. (2017). Curcumin Ameliorates Neuroinflammation, Neurodegeneration, and Memory Deficits in p25 Transgenic Mouse Model that Bears Hallmarks of Alzheimer’s Disease. J. Alzheimer’s Dis..

[35] Caruso Bavisotto C., Marino Gammazza A., Lo Cascio F., Mocciaro E., Vitale A.M., Vergilio G., Pace A., Cappello F., Campanella C., Palumbo Piccionello A. (2020). Curcumin Affects HSP60 Folding Activity and Levels in Neuroblastoma Cells. Int. J. Mol. Sci..

[36] Rodriguez Ospina S., Blazier D.M., Criado-Marrero M., Gould L.A., Gebru N.T., Beaulieu-Abdelahad D., Wang X., Remily-Wood E., Chaput D., Stevens S. (2022). Small Heat Shock Protein 22 Improves Cognition and Learning in the Tauopathic Brain. Int. J. Mol. Sci..

[37] Ringman J.M., Frautschy S.A., Teng E., Begum A.N., Bardens J., Beigi M., Gylys K.H., Badmaev V., Heath D.D., Apostolova L.G. (2012). Oral curcumin for Alzheimer’s disease: Tolerability and efficacy in a 24-week randomized, double blind, placebo-controlled study. Alzheimer’s Res. Ther..

[38] DiSilvestro R.A., Joseph E., Zhao S., Bomser J. (2012). Diverse effects of a low dose supplement of lipidated curcumin in healthy middle aged people. Nutr. J..

[39] Das S.S., Gopal P.M., Thomas J.V., Mohan M.C., Thomas S.C., Maliakel B.P., Krishnakumar I.M., Pulikkaparambil Sasidharan B.C. (2023). Influence of CurQfen®-curcumin on cognitive impairment: A randomized, double-blinded, placebo-controlled, 3-arm, 3-sequence comparative study. Front. Dement..

[40] Baum L., Lam C.W.K., Cheung S.K.-K., Kwok T., Lui V., Tsoh J., Lam L., Leung V., Hui E., Ng C. (2008). Six-Month Randomized, Placebo-Controlled, Double-Blind, Pilot Clinical Trial of Curcumin in Patients With Alzheimer Disease. J. Clin. Psychopharmacol..

[41] Freeman S.H., Raju S., Hyman B.T., Frosch M.P., Irizarry M.C. (2007). Plasma Aβ Levels Do Not Reflect Brain Aβ Levels. J. Neuropathol. Exp. Neurol..

[42] Toledo J.B., Vanderstichele H., Figurski M., Aisen P.S., Petersen R.C., Weiner M.W., Jack C.R., Jagust W., Decarli C., Toga A.W. (2011). Factors affecting Aβ plasma levels and their utility as biomarkers in ADNI. Acta Neuropathol..

[43] Giedraitis V., Sundelöf J., Irizarry M.C., Gårevik N., Hyman B.T., Wahlund L.-O., Ingelsson M., Lannfelt L. (2007). The normal equilibrium between CSF and plasma amyloid beta levels is disrupted in Alzheimer’s disease. Neurosci. Lett..

[44] Pascoal T.A., Aguzzoli C.S., Lussier F.Z., Crivelli L., Suemoto C.K., Fortea J., Rosa-Neto P., Zimmer E.R., Ferreira P.C.L., Bellaver B. (2024). Insights into the use of biomarkers in clinical trials in Alzheimer’s disease. eBioMedicine.

[45] Small G.W., Siddarth P., Li Z., Miller K.J., Ercoli L., Emerson N.D., Martinez J., Wong K.-P., Liu J., Merrill D.A. (2018). Memory and Brain Amyloid and Tau Effects of a Bioavailable Form of Curcumin in Non-Demented Adults: A Double-Blind, Placebo-Controlled 18-Month Trial. Am. J. Geriatr. Psychiatry.

[46] Thota R.N., Rosato J.I., Dias C.B., Burrows T.L., Martins R.N., Garg M.L. (2020). Dietary Supplementation with Curcumin Reduce Circulating Levels of Glycogen Synthase Kinase-3β and Islet Amyloid Polypeptide in Adults with High Risk of Type 2 Diabetes and Alzheimer’s Disease. Nutrients.

[47] Avgerinos K.I., Manolopoulos A., Ferrucci L., Kapogiannis D. (2024). Critical assessment of anti-amyloid-β monoclonal antibodies effects in Alzheimer’s disease: A systematic review and meta-analysis highlighting target engagement and clinical meaningfulness. Sci. Rep..

[48] Cummings J.L., Gonzalez M.I., Pritchard M.C., May P.C., Toledo-Sherman L.M., Harris G.A. (2023). The therapeutic landscape of tauopathies: Challenges and prospects. Alzheimer’s Res. Ther..

[49] Abdolahi M., Tafakhori A., Togha M., Okhovat A.A., Siassi F., Eshraghian M.R., Sedighiyan M., Djalali M., Mohammadzadeh Honarvar N., Djalali M. (2017). The synergistic effects of ω-3 fatty acids and nano-curcumin supplementation on tumor necrosis factor (TNF)-α gene expression and serum level in migraine patients. Immunogenetics.

[50] Cox K.H.M., White D.J., Pipingas A., Poorun K., Scholey A. (2020). Further Evidence of Benefits to Mood and Working Memory from Lipidated Curcumin in Healthy Older People: A 12-Week, Double-Blind, Placebo-Controlled, Partial Replication Study. Nutrients.

[51] den Haan J., Hart de Ruyter F.J., Lochocki B., Kroon M.A.G.M., Kemper E.M., Teunissen C.E., van Berckel B., Scheltens P., Hoozemans J.J., de Kreeke A.v. (2022). No difference in retinal fluorescence after oral curcumin intake in amyloid-proven AD cases compared to controls. Alzheimer’s Dement. Diagn. Assess. Dis. Monit..

[52] Fança-Berthon P., Tenon M., Bouter-Banon S.L., Manfré A., Maudet C., Dion A., Chevallier H., Laval J., van Breemen R.B. (2021). Pharmacokinetics of a Single Dose of Turmeric Curcuminoids Depends on Formulation: Results of a Human Crossover Study. J. Nutr..

[53] Kuszewski J.C., Howe P.R.C., Wong R.H.X. (2020). Evaluation of Cognitive Performance following Fish-Oil and Curcumin Supplementation in Middle-Aged and Older Adults with Overweight or Obesity. J. Nutr..

[54] Laksmidewi A.A.A., Soejitno A., Vania A., Mahendra I.N.B. (2024). Improving cognitive function with intermittent dose escalation of curcumin extract in chemotherapy-induced cognitive impairment patients: A randomized controlled trial. Adv. Tradit. Med..

[55] Panahi Y., Hosseini M.S., Khalili N., Naimi E., Majeed M., Sahebkar A. (2015). Antioxidant and anti-inflammatory effects of curcuminoid-piperine combination in subjects with metabolic syndrome: A randomized controlled trial and an updated meta-analysis. Clin. Nutr..

[56] Rainey-Smith S.R., Brown B.M., Sohrabi H.R., Shah T., Goozee K.G., Gupta V.B., Martins R.N. (2016). Curcumin and cognition: A randomised, placebo-controlled, double-blind study of community-dwelling older adults. Br. J. Nutr..

[57] Santos-Parker J.R., Strahler T.R., Bassett C.J., Bispham N.Z., Chonchol M.B., Seals D.R. (2017). Curcumin supplementation improves vascular endothelial function in healthy middle-aged and older adults by increasing nitric oxide bioavailability and reducing oxidative stress. Aging.

[58] Pizzino G., Irrera N., Cucinotta M., Pallio G., Mannino F., Arcoraci V., Squadrito F., Altavilla D., Bitto A. (2017). Oxidative Stress: Harms and Benefits for Human Health. Oxidative Med. Cell. Longev..

[59] Keller J.N., Schmitt F.A., Scheff S.W., Ding Q., Chen Q., Butterfield D.A., Markesbery W.R. (2005). Evidence of increased oxidative damage in subjects with mild cognitive impairment. Neurology.

[60] Palmer A.M., Burns M.A. (1994). Selective increase in lipid peroxidation in the inferior temporal cortex in Alzheimer’s disease. Brain Res..

[61] Ak T., Gülçin İ. (2008). Antioxidant and radical scavenging properties of curcumin. Chem.-Biol. Interact..

[62] Jovanovic S.V., Boone C.W., Steenken S., Trinoga, Manuela, Kaskey R.B. (2001). How curcumin works preferentially with water soluble antioxidants. J. Am. Chem. Soc..

[63] Galasko D.R., Peskind E., Clark C.M., Quinn J.F., Ringman J.M., Jicha G.A., Cotman C., Cottrell B., Montine T.J., Thomas R.G. (2012). Antioxidants for Alzheimer Disease: A Randomized Clinical Trial With Cerebrospinal Fluid Biomarker Measures. Arch. Neurol..

[64] Ahmed Nasef N., Loveday S.M., Golding M., Martins R.N., Shah T.M., Clarke M., Coad J., Moughan P.J., Garg M.L., Singh H. (2019). Food matrix and co-presence of turmeric compounds influence bioavailability of curcumin in healthy humans. Food Funct..

[65] Sakano K., Kawanishi S. (2002). Metal-mediated DNA damage induced by curcumin in the presence of human cytochrome P450 isozymes. Arch. Biochem. Biophys..

[66] Ahsan H., Hadi S.M. (1998). Strand scission in DNA induced by curcumin in the presence of Cu(II). Cancer Lett..

[67] Biswas J., Sinha D., Mukherjee S., Roy S., Siddiqi M., Roy M. (2010). Curcumin protects DNA damage in a chronically arsenic-exposed population of West Bengal. Hum. Exp. Toxicol..

[68] Kinney J.W., Bemiller S.M., Murtishaw A.S., Leisgang A.M., Salazar A.M., Lamb B.T. (2018). Inflammation as a central mechanism in Alzheimer’s disease. Alzheimer’s Dement. Transl. Res. Clin. Interv..

[69] Goldgaber D., Harris H.W., Hla T., Maciag T., Donnelly R.J., Jacobsen J.S., Vitek M.P., Gajdusek D.C. (1989). Interleukin 1 regulates synthesis of amyloid beta-protein precursor mRNA in human endothelial cells. Proc. Natl. Acad. Sci. USA.

[70] Huang P., Zheng N., Zhou H.-B., Huang J. (2020). Curcumin inhibits BACE1 expression through the interaction between ERβ and NFκB signaling pathway in SH-SY5Y cells. Mol. Cell. Biochem..

[71] Elbini-Dhouib I., Doghri R., Ellefi A., Degrach I., Srairi-Abid N., Gati A. (2021). Curcumin Attenuated Neurotoxicity in Sporadic Animal Model of Alzheimer’s Disease. Molecules.

[72] Lim G.P., Chu T., Yang F., Beech W., Frautschy S.A., Cole G.M. (2001). The Curry Spice Curcumin Reduces Oxidative Damage and Amyloid Pathology in an Alzheimer Transgenic Mouse. J. Neurosci..

[73] Heneka M.T., Carson M.J., Khoury J.E., Landreth G.E., Brosseron F., Feinstein D.L., Jacobs A.H., Wyss-Coray T., Vitorica J., Ransohoff R.M. (2015). Neuroinflammation in Alzheimer’s disease. Lancet Neurol..

[74] Rajesh Y., Kanneganti T.-D. (2022). Innate Immune Cell Death in Neuroinflammation and Alzheimer’s Disease. Cells.

[75] Jonsson T., Stefansson H., Steinberg S., Jonsdottir I., Jonsson Palmi V., Snaedal J., Bjornsson S., Huttenlocher J., Levey Allan I., Lah James J. (2013). Variant of TREM2 Associated with the Risk of Alzheimer’s Disease. N. Engl. J. Med..

[76] Rogers J., Luber-Narod J., Styren S.D., Civin W.H. (1988). Expression of immune system-associated antigens by cells of the human central nervous system: Relationship to the pathology of Alzheimer’s disease. Neurobiol. Aging.

[77] Halliday G.M., Cullen K.M., Kril J.J., Harding A.J., Harasty J. (1996). Glial fibrillary acidic protein (GFAP) immunohistochemistry in human cortex: A quantitative study using different antisera. Neurosci. Lett..

[78] Ambegaokar S.S., Wu L., Alamshahi K., Lau J., Jazayeri L., Chan S., Khanna P., Hsieh E., Timiras P.S. (2003). Curcumin inhibits dose-dependently and time-dependently neuroglial cell proliferation and growth. Neuroendocrinol. Lett..

[79] Zhang W., Sun M., Liu N., Li X., Sun J., Wang M. (2025). Curcumin ameliorates astrocyte inflammation through AXL in cuprizone-induced mice. Toxicol. Appl. Pharmacol..

[80] Seady M., Fróes F.T., Gonçalves C.A., Leite M.C. (2023). Curcumin modulates astrocyte function under basal and inflammatory conditions. Brain Res..

[81] Zhao X., van Praag H. (2020). Steps towards standardized quantification of adult neurogenesis. Nat. Commun..

[82] Kim S.J., Son T.G., Park H.R., Park M., Kim M.-S., Kim H.S., Chung H.Y., Mattson M.P., Lee J. (2008). Curcumin Stimulates Proliferation of Embryonic Neural Progenitor Cells and Neurogenesis in the Adult Hippocampus. J. Biol. Chem..

[83] He Y., Zhao Y., Lv R., Dong N., Wang X., Yu Q., Yue H.-m. (2024). Curcumin activates the Wnt/β-catenin signaling pathway to alleviate hippocampal neurogenesis abnormalities caused by intermittent hypoxia: A study based on network pharmacology and experimental verification. Int. Immunopharmacol..

[84] Lee Y., Park H.R., Lee J.Y., Kim J., Yang S., Lee C., Kim K., Kim H.S., Chang S.-C., Lee J. (2023). Low-dose curcumin enhances hippocampal neurogenesis and memory retention in young mice. Arch. Pharmacal Res..

[85] Li G., Wu Q., Wang C., Deng P., Li J., Zhai Z., Li Y. (2025). Curcumin reverses cognitive deficits through promoting neurogenesis and synapse plasticity via the upregulation of PSD95 and BDNF in mice. Sci. Rep..

[86] Dorostkar M.M., Zou C., Blazquez-Llorca L., Herms J. (2015). Analyzing dendritic spine pathology in Alzheimer’s disease: Problems and opportunities. Acta Neuropathol..

[87] Li B.-Z., Sumera A., Booker S.A., McCullagh E.A. (2023). Current Best Practices for Analysis of Dendritic Spine Morphology and Number in Neurodevelopmental Disorder Research. ACS Chem. Neurosci..

[88] González-Granillo A.E., Gnecco D., Díaz A., Garcés-Ramírez L., de la Cruz F., Juarez I., Morales-Medina J.C., Flores G. (2022). Curcumin induces cortico-hippocampal neuronal reshaping and memory improvements in aged mice. J. Chem. Neuroanat..

[89] Wang Y.-J., Pan M.-H., Cheng A.-L., Lin L.-I., Ho Y.-S., Hsieh C.-Y., Lin J.-K. (1997). Stability of curcumin in buffer solutions and characterization of its degradation products. J. Pharm. Biomed. Anal..

[90] Vaz G., Clementino A., Mitsou E., Ferrari E., Buttini F., Sissa C., Xenakis A., Sonvico F., Dora C.L. (2022). In Vitro Evaluation of Curcumin- and Quercetin-Loaded Nanoemulsions for Intranasal Administration: Effect of Surface Charge and Viscosity. Pharmaceutics.

[91] Koronyo Y., Biggs D., Barron E., Boyer D.S., Pearlman J.A., Au W.J., Kile S.J., Blanco A., Fuchs D.T., Ashfaq A. (2017). Retinal amyloid pathology and proof-of-concept imaging trial in Alzheimer’s disease. JCI Insight.

[92] Ireson C.R., Jones D.J., Orr S., Coughtrie M.W., Boocock D.J., Williams M.L., Farmer P.B., Steward W.P., Gescher A.J. (2002). Metabolism of the cancer chemopreventive agent curcumin in human and rat intestine. Cancer Epidemiol. Biomark. Prev..

[93] Josifovska S., Panov S., Hadzi-Petrushev N., Mitrokhin V., Kamkin A., Stojchevski R., Avtanski D., Mladenov M. (2023). Positive Tetrahydrocurcumin-Associated Brain-Related Metabolomic Implications. Molecules.

[94] Maiti P., Manna J., Thammathong J., Evans B., Dubey K.D., Banerjee S., Dunbar G.L. (2021). Tetrahydrocurcumin Has Similar Anti-Amyloid Properties as Curcumin: In Vitro Comparative Structure-Activity Studies. Antioxidants.

[95] Randino R., Grimaldi M., Persico M., De Santis A., Cini E., Cabri W., Riva A., D’Errico G., Fattorusso C., D’Ursi A.M. (2016). Investigating the Neuroprotective Effects of Turmeric Extract: Structural Interactions of β-Amyloid Peptide with Single Curcuminoids. Sci. Rep..

[96] Santoro A., Ricci A., Rodriquez M., Buonocore M., D’Ursi A.M. (2025). A Structural Effect of the Antioxidant Curcuminoids on the Aβ(1–42) Amyloid Peptide. Antioxidants.

[97] Arunkhamkaew S., Athipornchai A., Apiratikul N., Suksamrarn A., Ajavakom V. (2013). Novel racemic tetrahydrocurcuminoid dihydropyrimidinone analogues as potent acetylcholinesterase inhibitors. Bioorg. Med. Chem. Lett..

[98] Rahim-Mahdy H., Seifert R. (2026). A market and risk assessment of 125 turmeric supplements available in Australia, Germany, India, UK, and USA. Naunyn-Schmiedeberg’s Arch. Pharmacol..

[99] Casettari L., Illum L. (2014). Chitosan in nasal delivery systems for therapeutic drugs. J. Control. Release.

[100] Yen F.-L., Wu T.-H., Tzeng C.-W., Lin L.-T., Lin C.-C. (2010). Curcumin Nanoparticles Improve the Physicochemical Properties of Curcumin and Effectively Enhance Its Antioxidant and Antihepatoma Activities. J. Agric. Food Chem..

[101] Desai N. (2012). Challenges in Development of Nanoparticle-Based Therapeutics. AAPS J..

[102] Cheng K.K., Yeung C.F., Ho S.W., Chow S.F., Chow A.H.L., Baum L. (2013). Highly Stabilized Curcumin Nanoparticles Tested in an In Vitro Blood–Brain Barrier Model and in Alzheimer’s Disease Tg2576 Mice. AAPS J..

[103] Duan Z., Zhou W., He S., Wang W., Huang H., Yi L., Zhang R., Chen J., Zan X., You C. (2024). Intranasal Delivery of Curcumin Nanoparticles Improves Neuroinflammation and Neurological Deficits in Mice with Intracerebral Hemorrhage. Small Methods.

[104] Barbara R., Belletti D., Pederzoli F., Masoni M., Keller J., Ballestrazzi A., Vandelli M.A., Tosi G., Grabrucker A.M. (2017). Novel Curcumin loaded nanoparticles engineered for Blood-Brain Barrier crossing and able to disrupt Abeta aggregates. Int. J. Pharm..

[105] McClure R., Ong H., Janve V., Barton S., Zhu M., Li B., Dawes M., Jerome W.G., Anderson A., Massion P. (2017). Aerosol Delivery of Curcumin Reduced Amyloid-β Deposition and Improved Cognitive Performance in a Transgenic Model of Alzheimer’s Disease. J. Alzheimer’s Dis..

[106] Feng Q., Zhang X., Zhao X., Liu J., Wang Q., Yao Y., Xiao H., Zhu Y., Zhang W., Wang L. (2024). Intranasal Delivery of Pure Nanodrug Loaded Liposomes for Alzheimer’s Disease Treatment by Efficiently Regulating Microglial Polarization. Small.

[107] McClure R., Yanagisawa D., Stec D., Abdollahian D., Koktysh D., Xhillari D., Jaeger R., Stanwood G., Chekmenev E., Tooyama I. (2015). Inhalable Curcumin: Offering the Potential for Translation to Imaging and Treatment of Alzheimer’s Disease. J. Alzheimer’s Dis..

[108] DiMauro T.M., DePuy Spine LLC, DePuy Synthes Products Inc (2013). Curcumin Derivatives. U.S. Patent.

[109] Aggarwal B., Knight J., Research Development Foundation (2005). Aerosol Delivery of Curcumin. U.S. Patent.

