# Supplementary material for: Translational Feasibility of Curcumin for Treatment of Alzheimer’s Disease: A Critical Appraisal of Clinical Challenges
Source: Antioxidants (Basel). 2026 May 18;15(5):638. doi: 10.3390/antiox15050638 (PMC13203765; doi:10.3390/antiox15050638)
Supplement: Supplementary file 1 [file antioxidants-15-00638-s001.zip › antioxidants-4263340-supplementary.pdf]

Table S1: Preclinical trial summary table

Study ID	Preclinical Model	Intervention	Duration	Primary Outcome
Caesar *et al.* 2012 [19]	Drosophila; 20 days old; UAS-Aβ1–40 (n = 100), UAS-Aβ1–40 (n = 100), UAS-Aβ1–42;UAS-Aβ1–42 (n = 100), UAS-Aβ1–42 E22G (n = 100), UAS-tau (n = 60-100), control (n = 100) *	Curcumin in yeast paste; 0.0001%, 0.001%, 0.01% (w/w); oral	20 days	Formulating a pharmacological treatment for Drosophila AD models
Cheng *et al.* 2012 [102]	Mouse; Tg2576; 9 months old; nanocurcumin (n = 20), curcumin (n = 20), control (n = 20)	23mg/kg curcumin, nanocurcumin or water; oral	21 days	Develop an oral nano formulation of curcumin which details the treatment effect
Duan *et al.* 2024 [103]	Mouse; C57BL/6J; 6-8 weeks old; ICH model established via injecting 0.15 U/μL, 0.5 μL collagenase or 30μL autologous arterial blood per mouse in the right basal ganglia	mPEG-PCL 150mg/ml control (intravenous); curcumin nanoparticles 15mg/ml (intranasal); free curcumin 16mg/ml (intravenous)	7 days	Study the therapeutic effects of intranasal curcumin nanoparticles in regulating neuroinflammation
ElBini-Dhouib *et al.* 2021 [71]	Rat; Wistar albino; negative control (n = 6), positive control receiving 100mg/kg $AlCl_3$ (n = 18), curcumin group receiving 100mg/kg $AlCl_3$ (n = 6)	100mg/kg curcumin dissolved in 1mL of corn oil via intragastric delivery; control given no treatment; intragastric	150 days	Assessing the ability of curcumin to induce neuroprotective and recovery effects in a rat model of neurotoxicity mimicking sporadic AD
Feng *et al.* 2024 [106]	Mouse; C57BL/8J, APP/PS1; 7 months old; n numbers unclear	Intranasal saline control; curcumin once daily 7.36 mg/mL via intranasal nanoparticles	30 days	Assess how intranasal, pure nanodrug loaded liposomes of curcumin effect AD mouse models
Lee *et al.* 2023 [84]	Mouse; C57BL/6; 4 weeks old; curcumin (n = 15-30), control (n = 15-30)	Curcumin dissolved in PBS at 0.4, 2, 10mg/kg; same volume of PBS administered in control; oral	14 days	Examining whether subchronic oral administration of curcumin affects hippocampal neurogenesis
Lim *et al.* 2001 [72]	Mouse; APPsw; 10 months old; tg low dose curcumin (n = 9), tg high dose curcumin (n = 6), tg control (n = 8), wt control (n = 5)	160 ppm curcumin chow; 5000 ppm curcumin chow; control chow; oral	180 days	Testing curcumin’s ability to inhibit inflammatory and oxidative damage which occurs in response to amyloid burden
Lou *et al.* 2024 [29]	Mouse; C57BL/6J; 2 months old; Aβ1–42 ICV (n = 48), Aβ1–42 + MSAB ICV (n = 48), Aβ1–42 + MSAB ICV + curcumin (n = 48), Aβ1–42 + Fc ICV + curcumin (N = 48)	0.3nmol LY294002 and 0.6nmol MSAB ICV injection 30 minutes before intragastric delivery of 100mg/kg curcumin; controls given saline including 0.5% dimethyl sulfoxide ICV	14 days	Exploring whether curcumin can improve adult hippocampal neurogenesis through the AHN Wnt/β-catenin and BDNF pathway in AD model mice
Ma *et al.* 2012 [33]	Mouse; C57BL/6J; 15-16 months old; htau control (n = 11), htau curcumin (n = 9), wt control (n = 10)	500ppm curcumin solid lipid nanoparticle Longvida in breeding chow; breeding chow control; oral	28 days	Whether curcumin diet is significantly different to a normal

				diet in tau AD mice modelling human AD pathology
McClure *et al.* 2016 [105]	Mouse; C57BL/6, 5XFAD; 6 weeks old; treatment transgenic group (n = 10), control tg group (n = 10), control wt group (n = 10)	5mg/kg nebulized curcumin three times a week for 18 weeks;	183 days	Aim to show that their curcumin formulation can have a significant therapeutic effect and cross the blood brain barrier
Yang *et al.* 2005 [21]	Mouse; APPsw Tg2576; 17 months old; curcumin diet (n ≥ 9), control diet (n ≥ 8);	Curcumin, 500ppm; safflower oil-based control; oral	510 days	Demonstrating curcumin's ability to bind to amyloid and inhibit aggregation and the fibrillation pathway with dosing at acceptable levels
Zhang *et al.* 2025 [79]	Mouse; C57BL/6; 6-8 weeks; wt control (n =6), wt curcumin (n = 6), wt CPZ (n = 6), wt curcumin and CPZ (n =6), $AXL^{-/-}$ control (n = 6), $AXL^{-/-}$ and CPZ (n = 6), $AXL^{-/-}$ curcumin and CPZ (n = 6)	0.2% (w/w) CPZ mixed with AIN-76A rodent diet for 5 weeks to induce myelin atrophy in CPZ groups; 200/mg curcumin with 0.5% DMSO	21 days	Exploring whether curcumin regulates astrocyte-mediated inflammatory neuroinflammation and demyelination through the AXL receptor
